# Simultaneous Determination of Chlorogenic Acid Isomers and Metabolites in Rat Plasma Using LC-MS/MS and Its Application to A Pharmacokinetic Study Following Oral Administration of *Stauntonia Hexaphylla* Leaf Extract (YRA-1909) to Rats

**DOI:** 10.3390/pharmaceutics10030143

**Published:** 2018-09-02

**Authors:** Won-Gu Choi, Ju-Hyun Kim, Dong Kyun Kim, Yongnam Lee, Ji Seok Yoo, Dae Hee Shin, Hye Suk Lee

**Affiliations:** 1 BK21 PLUS Team for Creative Leader Program for Pharmacomics-based Future Pharmacy and Drug Metabolism and Bioanalysis Laboratory, College of Pharmacy, The Catholic University of Korea, Bucheon 420-743, Korea; cwg0222@catholic.ac.kr (W.-G.C.); noraekajoa@gmail.com (J.-H.K.); kdk3124@catholic.ac.kr (D.K.K.); 2 College of Pharmacy, Yeungnam University, Gyeongsan 38541, Korea; 3 Central R&D Institute, YUNGJIN PHARM. CO., LTD., Suwon 16229, Korea; nami0209@yungjin.co.kr (Y.L.); jsyoo@yungjin.co.kr (J.S.Y.); sdh580509@gmail.com (D.H.S.)

**Keywords:** *Stauntonia hexaphylla* leaf extract, YRA-1909, pharmacokinetics, chlorogenic acid, neochlorogenic acid, cryptochlorogenic acid, caffeic acid, caffeic acid *O*-glucuronides, LC-MS/MS

## Abstract

*Stauntonia hexaphylla* leaf extract (YRA-1909), which is widely used for the antirheumatic properties, has been under phase 2 clinical trials in patients with rheumatoid arthritis since April 2017. Liquid chromatography-tandem mass spectrometric method while using liquid–liquid extraction with ethyl acetate was validated for the simultaneous determination of the major active components of YRA-1909, including chlorogenic acid (CGA), neochlorogenic acid (NCGA), cryptochlorogenic acid (CCGA), and their metabolites (i.e., caffeic acid (CA), caffeic acid 3-*O*-glucuronide (CA-3-G), caffeic acid 4-*O*-glucuronide (CA-4-G), and ferulic acid (FA)) in rat plasma and applied to a pharmacokinetic study of YRA-1909 in rats. Seven analytes were separated on Halo C18 while using gradient elution of formic acid and methanol, and then quantified in selected reaction monitoring mode whle using negative electrospray ionization. Following oral administration of YRA-1909 at doses of 25, 50, and 100 mg/kg to male Sprague-Dawley rats, CGA, NCGA, and CCGA were rapidly absorbed and metabolized to CA, CA-3-G, and CA-4-G. The area under the plasma concentration-time curve (AUC_last_) of CGA, NCGA, CCGA, and three metabolites linearly increased as the YRA-1909 dose increased. Other pharmacokinetic parameters were comparable among three doses studied. AUC_last_ values for CA, CA-3-G, and CA-4-G exceeded those for CGA, NCGA, and CCGA.

## 1. Introduction

**Figure 1:**
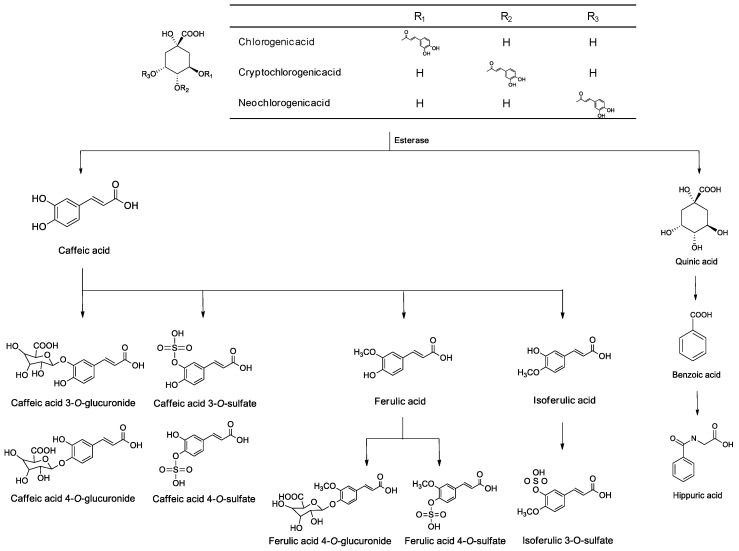
Metabolic pathways of chlorogenic acid, neochlorogenic acid, and cryptochlorogenic acid in rats and humans.

*Stauntonia hexaphylla* (Lardizabalaceae) is widely distributed throughout Korea, Japan, and China, and is a popular herbal supplement in Korean and Chinese folk medicine due to its analgesic, sedative, and diuretic properties [1,2]. It exhibits anti-osteoporosis [3], antidiabetic activity [4], and anti-inflammatory effects in carrageenan-induced paw edema rats [5]. *Stauntonia hexaphylla* leaf extract, YRA-1909, is associated with the antirheumatic activity [6] and has been undergoing in phase 2 clinical trials in the Republic of Korea since April 2017 to evaluate its safety and efficacy for treating patients suffering from rheumatoid arthritis (ClinicalTrials.gov identifier NCT03275025) [7]. Chlorogenic acid (CGA), neochlorogenic acid (NCGA), and cryptochlorogenic acid (CCGA) have been identified as the active ingredients in *Stauntonia hexaphylla* [4,5] and YRA-1909. CGA and NCGA are associated with various pharmacological properties, including antioxidant [8], anti-cancer [9], anti-virus [10], anti-coagulant, and anti-thrombotic properties [11]. CGA, NCGA, and CCGA are metabolized to caffeic acid (CA) and quinic acid by esterase [12,13,14,15]. CA is further metabolized to caffeic acid 3-*O*-glucuronide (CA-3-G) and caffeic acid 4-*O*-glucuronide (CA-4-G) by uridine-5′-diphosphate-glucuronosyltransferase (UGT) 1A1 and 1A9 enzymes, caffeic acid 3- and 4-*O*-sulfate by sulfotransferase (SULT) 1A1 and 1E1 enzymes, and ferulic acid (FA) and isoferulic acid by catechol-*O*-methyltransferase (COMT), which are metabolized to FA 4-*O*-glucuronide, FA 4-*O*-sulfate, and isoferulic acid 3-*O*-sulfate (Figure 1) [12,13,14,15,16]. CA and FA are active metabolites that are associated with various biological activities, including antioxidant [17], anti-mutagenic [18], and anti-cancer [19,20] effects. 

Pharmacokinetic evaluation of herbal drugs is helpful for determining dosage regimens and interpreting the pharmacological effects under clinical conditions. However, typical pharmacokinetic studies can be difficult to apply to herbal drugs that consist of multiple constituents, with frequently unidentified pharmacologically active components. As such, simultaneous determination of the various potential active constituents, major constituents, and/or their metabolites is recommended for the satisfactory pharmacokinetic evaluation of herbal drugs [21,22,23].

Several studies have applied liquid chromatography-mass spectrometry (LC-MS) or tandem mass spectrometry (LC-MS/MS) methods for the simultaneous determination of CGA, NCGA, CCGA, CA, or FA in human and rat plasma (50–500 μL) while using liquid–liquid extraction [24,25,26,27], or solid phase extraction [28], protein precipitation [29], or protein precipitation with disperse solid phase extraction [30]. The LC-MS has also been used in pharmacokinetic studies of these acids following the administration of herbal drugs to rats and humans. For example, one such study used the LC-MS/MS method to simultaneously determine phenolic acids and their metabolites in human plasma following the ingestion of instant coffee containing various chlorogenic and phenolic acids; the study used protein precipitation to prepare samples [31]. The negative [24,26,28,29,30,31] and positive [25,27] electrospray ionization (ESI) modes were used for the ionization of phenolic acids in mass spectrometry.

There are currently no reports on the pharmacokinetics of YRA-1909. In this study, we developed a sensitive and selective LC-MS/MS method for evaluating the pharmacokinetics of the active constituents of YRA-1909 (i.e., CGA, NCGA, and CCGA) and their active metabolites (i.e., CA, CA-3-G, CA-4-G, and FA) after the oral administration of YRA-1909 at doses of 25, 50, and 100 mg/kg to the rats. 

## 2. Materials and Methods

### 2.1. Materials

CGA (purity, 98.1%) was obtained from Hunan Yuanhang Biology Technology Co., Ltd (Changsha, China). CCGA (purity, 99.7%) was obtained from Dailan Meilun Biotech Co., Ltd (Dailan, China). NCGA (purity, 98.0%), CA (purity, 98.0%), FA (purity, 99.0%), dimethyl sulfoxide, and formic acid were purchased from Sigma Aldrich Co. (St. Louis, MO, USA). CA-3-G (purity, 98.1%), CA-4-G (purity, 98.0%), and ferulic acid-d_3_ (FA-d_3_)(purity, 98.0%; used as an internal standard) were obtained from Toronto Research Chemicals Inc. (Toronto, Canada). Water and methanol (LC-MS grade) were supplied from Burdick and Jackson Inc. (Muskegon, MI, USA). All the other chemicals used were of the highest quality available.

*Stauntonia hexaphylla* leaves were collected from cultivated fields, in accordance with Good Agricultural Practice guidelines, in Jangheung (Jeonnam, Korea). YRA-1909 was prepared by extracting *Stauntonia hexalphylla* leaves with distilled water, followed by purification using column chromatography. YRA-1909 was produced by KGCYebon (Chungju, Korea) according to the protocol recommended by the International Conference on Harmonization and Good Manufacturing Practice. The contents of CGA, NCGA, and CCGA in YRA-1909 (batch no.: YR-1001) were 4.74, 1.10, and 3.49 mg/g, respectively, as quantified while using high-performance liquid chromatographic method for quality control developed by YUNGJIN PHARM CO., LTD (Suwon, Korea). CA and FA were not detected in YRA-1909.

### 2.2. Preparation of Clibration Standards and Quality Control Samples

Each standard stock solution was prepared separately by dissolving CGA, NCGA, CCGA, and their four metabolites (1 mg each) in 1 mL dimethyl sulfoxide. A mixed standard stock solution was prepared by mixing each stock solution to final concentrations of 10 μg/mL for CGA and NCGA, 50 μg/mL for CCGA, CA, CA-3-G, and CA-4-G, and 250 μg/mL for FA. Working standard solutions of the seven analytes were prepared by diluting a mixed standard stock solution with methanol. The internal standard working solution (FA-d_3_, 20 μg/mL) was prepared by diluting an aliquot of stock solution with methanol. All such standard solutions were stored at 4 °C in darkness for four weeks. 

Rat plasma calibration standards were prepared at eight concentration levels by adding 2 μL working standard solution to 50 μL of drug-free rat plasma: 0.5, 1.0, 2.0, 5.0, 10.0, 25.0, 50,0, 100, and 200 ng/mL for CGA and NCGA, 2.5, 5.0, 10.0, 25.0, 50,0, 250, 500, and 1000 ng/mL for CCGA, CA, CA-3-G, and CA-4-G, and 12.5, 25.0, 50.0, 125, 250, 1250, 3750, and 5000 ng/mL for FA, respectively. 5 μL of 5% formic acid was added and vortex-mixed to improve the plasma stability of CGA, CCGA, and NCGA [24,32]. Quality control (QC) samples were prepared at the concentrations of 1.5, 25, and 160 ng/mL for CGA and NCGA, 7.5, 125, and 800 ng/mL for CCGA, CA, CA-3-G, and CA-4-G, and 37.5, 625, and 4000 ng/mL for FA in drug-free rat plasma and then stored at −80 °C until analysis.

### 2.3. Sample Preparation

50 μL aliquot of blank rat plasma, calibration standards, and QC samples stored on ice were vortex-mixed with 5 μL FA-d_3_ in methanol (20 μg/mL), 100 μL 1 M hydrochloric acid, and 600 μL ethyl acetate for 2 min. Following centrifugation at 13000 rpm for 8 min, 475 μL the supernatant was transferred into a new polypropylene tube. The aqueous layer was extracted once more with 600 μL ethyl acetate, and the supernatants were transferred after centrifugation. The organic layer was evaporated to dryness at 35 °C for 20 min while using a vacuum evaporator. The residues were dissolved in 50 μL 10% methanol and centrifuged. An aliquot (3 μL) was injected onto the LC-MS/MS system for analyses.

### 2.4. LC-MS/MS Analysis

An ultra-performance liquid chromatograph, Agilent 1290, coupled with Agilent 6495 tandem mass spectrometer (Agilent Technologies, Wilmington, DE, USA) was used. Chromatographic separation was performed on a Halo C_18_ column (2.7 μm, 2.1 mm i.d. × 100 mm, Advanced Material Technology, Wilmington, DE, USA) while using a gradient elution of 0.1% formic acid in water (mobile phase A) and 0.1% formic acid in methanol (mobile phase B) at a flow rate of 0.3 mL/min, as follows: 10% mobile phase B for 0.5 min, 10% to 25% mobile phase B for 5.5 min, 25% to 37% mobile phase B for 1 min, 37% mobile phase B for 2 min, 37% to 10% mobile phase B for 0.1 min, and 10% mobile phase B for 2.4 min. The column and autosampler tray were maintained at 35 °C and 4 °C, respectively. The ESI source settings for the ionization of the analytes in negative mode were as follows: gas temperature, 230 °C; gas flow, 19 L/min; Nebulizer, 45 psi; sheath gas temperature, 400 °C; sheath gas flow, 10 L/min; capillary voltage, 3000 V; nozzle voltage, 1000 V. Fragmentation of analytes was performed at collision energy of 14 eV for CGA, NCGA, and CCGA, 8 eV for CA, 20 eV for CA-3-G and CA-4-G, 12 eV for FA, and 10 eV for FA-d_3_, respectively, while using nitrogen gas as a collision gas at a pressure of 2 bar on the instrument. Selected reaction monitoring (SRM) mode was employed for the quantification: *m/z* 352.8 → 191.0 for CGA, NCGA, and CCGA; *m/z* 179.1 → 135.0 for CA; *m/z* 355.0 → 178.9 for CA-3-G and CA-4-G; *m/z* 192.9 → 133.9 for FA; *m/z* 196.1 → 134.0 for FA-d_3_. Mass Hunter software (Agilent Technologies) was used for LC-MS/MS system control and data processing.

### 2.5. Method Validation

Method validation was performed according to the FDA Guidance on Bioanalytical Method Validation. For the evaluation of intra-and inter-day precision and accuracy, we analyzed batches of calibration standards and QC samples in five replicates on three different days, as follows: 0.5, 1.5, 25, and 160 ng/mL for CGA and NCGA, 2.5, 7.5, 125, and 800 ng/mL for CCGA, CA, CA-3-G, and CA-4-G, and 12.5, 37.5, 625, and 4000 ng/mL for FA were analyzed. Accuracy was defined as relative error (RE, %) of the measured mean value deviated from the nominal value, and precision was defined as the coefficient of variation (CV, %) of the measured concentration.

The stability of each of the seven analytes in rat plasma was evaluated by analyzing low and high QC samples in triplicate: post-preparation sample stability in the autosampler at 4 °C for 24 h, short-term storage stability following storage of plasma samples on ice for 2 h, long-term storage stability following the storage of plasma samples at −80 °C for 28 days, and three freeze-thaw cycles. 

The matrix effect for each analyte was assessed by comparing the mean peak areas of the analytes that were spiked after extraction into blank plasma extracts originating from six different rats to mean peak areas for neat solutions of the analytes at 1.5, 25, and 160 ng/mL for CGA and NCGA, 7.5, 125, and 800 ng/mL for CCGA, CA, CA-3-G, and CA-4-G, and 37.5, 625, and 4000 ng/mL for FA. The recoveries of each analyte were determined by comparing the mean peak areas of the extract of analyte-spiked plasma with those of the analytes spiked post-extraction into six different blank plasma extracts at three concentration levels.

### 2.6. Pharmacokinetic Study of YRA-1909 in Rats

This validated method was applied to the pharmacokinetic study of CGA, NCGA, CCGA, and their metabolites, such as CA, CA-3-G, CA-4-G, and FA after a single oral administration of YRA-1909 at doses of 25 (*n* = 6), 50 (*n* = 12), and 100 mg/kg (*n* = 6) and repeated oral administration of 50 mg/kg YRA-1909 for seven days (*n* = 6) to male Sprague-Dawley (SD) rats (body weight, 220–260 g, Samtako Co., Osan, Korea). The effective oral doses of YRA-1909 on preclinical pharmacology studies were 25–100 mg/kg in rats (internal reports of YUNGJIN PHARM CO., LTD). The study protocol was approved by the Institutional Animal Care and Use Committee of The Catholic University of Korea (Approval No. 2015–015). The animals were kept in plastic cages with unlimited access to standard rat diet (Samtako Co.) and water before the experiment. Animals were maintained at a temperature of 23 ± 2 °C with a 12 h light/dark cycle and relative humidity of 50 ± 10%. The rats were anesthetized while using isoflurane and they were cannulated with polyethylene tubing (PE-50, Nastsume Co., Tokyo, Japan) in the jugular vein for blood sampling. Each rat was housed individually in a metabolic cage and permitted a recovery time of one day following anesthesia prior to the study’s commencement. During the recovery period, rats were fasted but let free access to water and were not restrained at any time. To prevent blood clotting, each catheter was flushed with heparin in a physiological saline solution (10 U/mL). YRA-1909 was dissolved in purified water and administered to the rats at doses of 25, 50, and 100 mg/kg (equivalent to 1.2, 2.4, and 4.8 mg/kg CGA; 0.275, 0.55, and 1.10 mg/kg NCGA; 0.87, 1.74, and 3.48 mg/kg CCGA). Blood samples (approximately 200 μL) were collected before (control) and 0.083, 0.25, 0.5, 1, 1.5, 2, 3, 4, 6, 8, and 24 h after drug administration. Plasma samples were harvested by centrifugation at 3000 g for 5 min at 4 °C and 50 μL plasma samples were immediately collected and vortex-mixed with 5 μL 5% formic acid. The samples were stored at −80 °C until analyses. 

Pharmacokinetic parameters were analyzed while using noncompartment analysis (WinNonlin, Pharsight, Mountain View, CA, USA), including the area under the plasma concentration-time curve during the period of observation (AUC_last_), the terminal half-life (t_1/2_), and mean residence time (MRT). The peak plasma concentration (C_max_) and the time to reach C_max_ (T_max_) were directly obtained from the experimental data. All data are expressed as the means ± standard deviations (S.D.).

## 3. Results

### 3.1. LC-MS/MS Analysis

**Figure 2:**
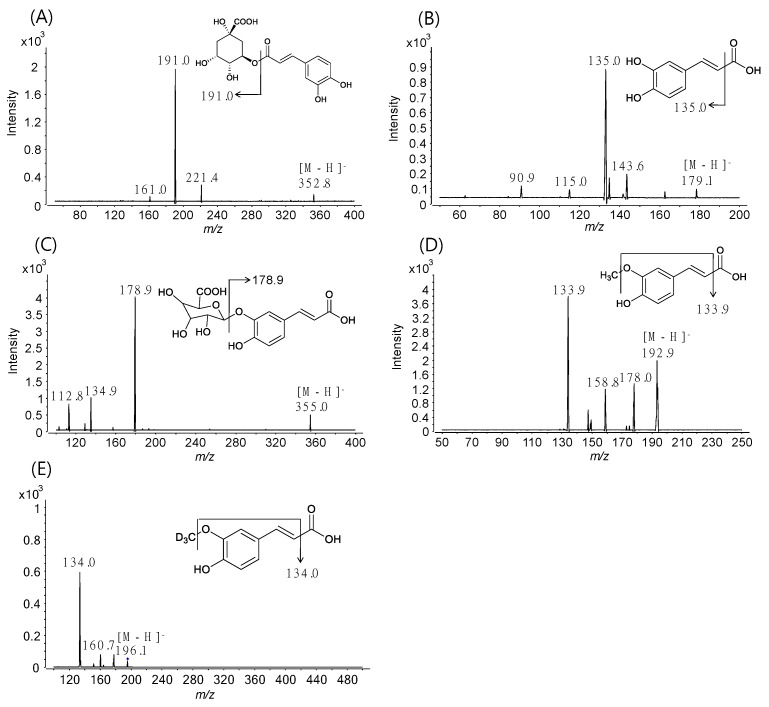
Product ion spectra of (**A**) chlorogenic acid (CGA), (**B**) caffeic acid (CA), (**C**) CA-3-G, (**D**) ferulic acid (FA), and (**E**) FA-d_3_ (IS).

For the simultaneous quantification of CGA, NCGA, CCGA, CA, CA-3-G, CA-4-G, and FA, MS/MS parameters for all analytes were optimized by the flow-injection method to achieve maximum sensitivity, and SRM transitions of the precursor ion ([M − H]^−^) to the intense product ion were used for data acquisition owing to the high selectivity and sensitivity (Figure 2) [24,28,29,30,31].

**Figure 3:**
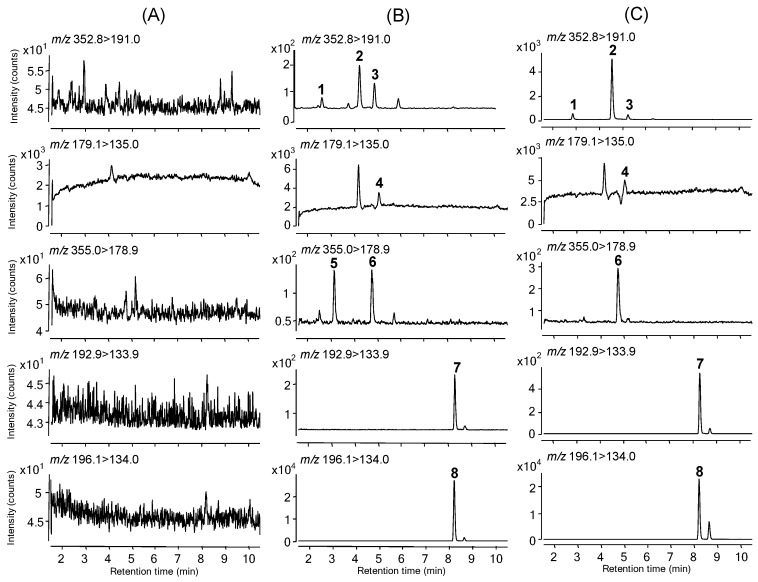
Selected reaction monitoring chromatograms of (**A**) rat blank plasma, (**B**) rat plasma spiked with CGA (0.5 ng/mL), NCGA (0.5 ng/mL), CCGA (2.5 ng/mL), CA (2.5 ng/mL), CA-3-G (2.5 ng/mL), CA-4-G (2.5 ng/mL), and FA (12.5 ng/mL) at lower limits of quantification (LLOQ) level, and (**C**) rat plasma obtained 5 min after oral administration of YRA-1909 at a dose of 100 mg/kg to a male SD rat. 1, NCGA; 2, CGA; 3, CCGA; 4, CA; 5, CA-4-G; 6, CA-3-G; 7, FA; 8, FA-d_3_.

The retention and base-line separation data are shown in Figure 3B. Analysis of blank plasma samples that were obtained from 30 different rats revealed no significant interference peaks in the retention times across all analytes, which indicates the selectivity of the present method (Figure 3A). Figure 3C illustrates representative SRM chromatograms of a plasma sample obtained 5 min after oral administration of YRA-1909 at a 100 mg/kg dosage in a male SD rat. 

### 3.2. Method Validation

Calibration curves were linear at concentration ranges of 0.5–200 ng/mL for CGA and NCGA, 12.5–5000 ng/mL for FA, and 2.5–1000 ng/mL for CCGA, CA, CA-3-G, and CA-4-G in rat plasma with coefficients of determination ≥0.9959 using linear regression analysis with a weighting of 1/concentration (Table 1). The RE and CV values of the calculated concentrations were less than ±15% and 15%, respectively, for all eight calibration points. The low CV values (≤12.2%) for the regression line slopes of the seven analytes indicated the repeatability of the method. 

The lower limits of quantification (LLOQ), the lowest amounts of the analytes in rat plasma sample that can be quantified with S/N ratio >5 as well as both CV and RE within 20% of nominal concentration, were 0.5 ng/mL for CGA and NCGA, 12.5 ng/mL for FA, and 2.5 ng/mL for CCGA, CA, CA-3-G, and CA-4-G in rat plasma (Table 1).

The intra-day and inter-day precision and accuracy values for QC samples are presented in Table 1. The CV values of the seven analytes ranged from 2.4% to 12.5% at low, medium, and high QC levels and from 4.1% to 18.1% at LLOQ QC levels. The RE values ranged from −10.0% to 10.4% at low, medium, and high QC levels and from −8.8% to 16.0% at LLOQ QC levels. These results confirm the method’s acceptable accuracy and precision levels.

A protein precipitation technique while using acetonitrile and methanol was examined as the sample preparation procedure for rat plasma but it was inadequate due to severe matrix effects. The recovery and matrix effects of all analytes in rat plasma were evaluated by extracting acidified rat plasma with ethyl acetate [25], ethyl acetate/ether [24], ethyl acetate/hexane [26], and methyl *ter*-butyl ether. Liquid–liquid extraction using ethyl acetate as the extraction solvent resulted in a higher degree of recovery and fewer matrix effects for all analytes (Table 2): the matrix effects and CV values were 86.5−98.0% and ≤11.8%, respectively. The recovery levels using liquid–liquid extraction at low, medium, and high QC levels are shown in Table 2. Based on the results, liquid–liquid extraction using ethyl acetate after acidification of rat plasma is deemed to be acceptable for the sample preparation procedure.

The processing (freeze-thaw, long-term storage at −80°C, and short-term storage on ice) and post-preparation stabilities of each analyte were evaluated; such processes had negligible effects on the stability of samples (Table 3).

### 3.3. Pharmacokinetics of YRA-1909 in Male SD Rats

**Figure 4:**
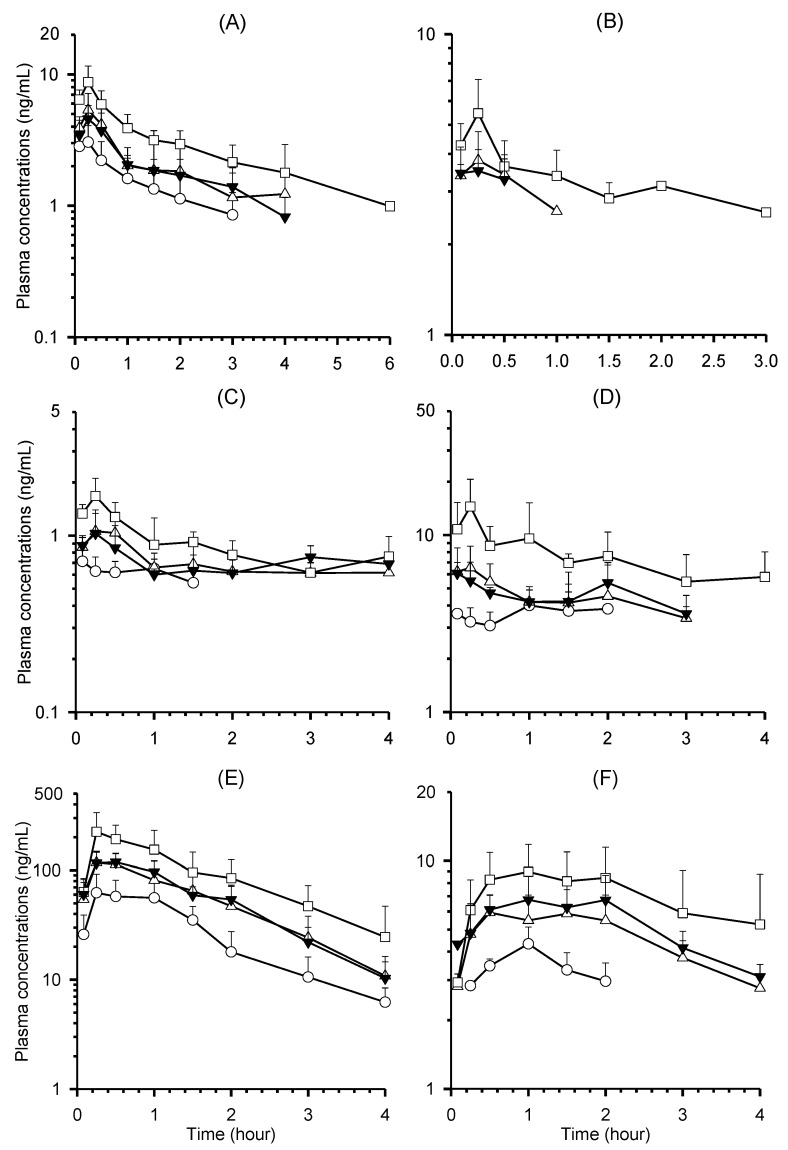
Mean plasma concentration-time profiles of (**A**) CGA, (**B**) CCGA, (**C**) neochlorogenic acid (NCGA), (**D**) CA, (**E**) CA-3-G, and (**F**) CA-4-G after a single oral administration of YRA-1909 at the doses of 25 (◯, *n* = 6), 50 (△, *n* = 12), and 100 (☐, *n* = 6) mg/kg (equivalent to 1.2, 2.4, and 4.8 mg/kg CGA; 0.275, 0.55, and 1.10 mg/kg NCGA; 0.87, 1.74 and 3.48 mg/kg CCGA) and repeated administration of 50 mg/kg YRA-1909 once/day for seven days (▼, *n* = 6) in male rats. Each point represents mean ± SD.

Mean plasma concentration-time profiles of CGA, NCGA, CCGA, CA, CA-3-G, and CA-4-G following a single oral administration of YRA-1909 at doses of 25, 50, and 100 mg/kg to male SD rats are shown in Figure 4. Plasma concentrations of CGA, NCGA, CA, CA-3-G, and CA-4-G were below LLOQ 6, 4, 4, 4, and 4 h after oral administration of YRA-1909 at a dosage of 100 mg/kg. Plasma concentrations of CCGA were under LLOQ (2.5 ng/mL) following 25 mg/kg dose (equivalent to 0.87 mg/kg CCGA), and 1 and 3 h after administration of 50 and 100 mg/kg doses of YRA-1909, respectively. Plasma concentrations of FA, which is a metabolite, were under LLOQ (12.5 ng/mL), except in samples taken 0.083 and/or 0.25 h after oral administration of 50 and 100 mg/kg YRA-1909. Therefore, the pharmacokinetic parameters of FA were not calculated. The relevant pharmacokinetic parameters of all other analytes are shown in Table 4. 

No significant differences were observed among plasma concentrations and pharmacokinetic parameters of CGA, NCGA, CCGA, CA, CA-3-G, and CA-4-G between single and seven-day repeated administration of 50 mg/kg YRA-1909 (Figure 4, Table 4).

## 4. Discussion

We developed a LC-MS/MS method for the simultaneous determination of CGA, NCGA, CCGA, and their metabolites, such as CA, CA-3-G, CA-4-G, and FA in rat plasma. A gradient elution of formic acid and methanol, as the mobile phase, resulted in less peak tailing and an increase in the ionization efficiency of the seven analytes, when compared to previously reported gradient elution of acetic acid-acetonitrile used as the mobile phase for the analyses of phenolic acids, sulfates, and glucuronides [31]. For sample preparation, liquid–liquid extraction using ethyl acetate at acidic pH resulted in smaller sample volume (50 vs. 100 μL), superior sensitivity (0.5 vs. 1.77 ng/mL for CGA and NCGA), and reduced matrix effects compared to the protein precipitation technique [31]. We successfully applied the present method to study pharmacokinetics of CGA, NCGA, CCGA, and their metabolites after oral administration of YRA-1909 (Figure 4, Table 4). 

CGA, NCGA, and CCGA were rapidly absorbed after oral administration of YRA-1909 at doses of 25, 50, and 100 mg/kg; each was detected at the first blood sampling time point (5 min) with rapid T_max_, 0.17–0.25 h for all three doses (Table 4). The AUC_last_ values for CGA and NCGA increased linearly as the oral dose increased. The dose-normalized (based on 25 mg/kg YRA-1909) AUC_last_ of CGA and NCGA were comparable among the doses studied: 4.29 ± 1.92, 4.08 ± 1.82, and 3.60 ± 1.27 ng·h/mL for CGA, respectively, and 0.81 ± 0.22, 0.81 ± 0.45, and 0.85 ± 0.49 ng·h/mL for NCGA in 25, 50, and 100 mg/kg YRA-1909, respectively. Moreover, the slopes between the log AUC_last_ and log dose of CGA and NCGA were close to 1. Plasma concentrations of CCGA were less than LLOQ (2.5 ng/mL) in the group that was administered a 25 mg/kg dose of YRA-1909, but the dose-normalized (based on 50 mg/kg YRA-1909) AUC_last_ values of CCGA were also comparable: 4.15 ± 4.10 and 3.13 ± 2.35 ng·h/mL for 50 and 100 mg/kg YRA-1909, respectively. 

CGA, NCGA, and CCGA were absorbed by passive diffusion and hydrolyzed to CA by gastric esterase, and then, CA was metabolized to ferulic acid by COMT [13]. The bioavailability and urinary recovery of CGA or CA were very low after oral administration of CGA or CA in rats due to extensive metabolism [14,15,31]. After oral administration of YRA-1909 at doses of 25, 50, and 100 mg/kg, CA, CA-3-G, CA-4-G, and FA, which were reported as the major metabolites of CGA, CCGA, and NCGA (Figure 1) [12,13,14,15], were determined in plasma that was obtained after oral administration of YRA-1909 (Figure 3, Table 4). AUC_last_ and C_max_ values of CA, CA-3-G, and CA-4-G linearly increased with an increase in YRA-1909 dose (Figure 4, Table 4). For CA, dose-normalized (based on 25 mg/kg YRA-1909) AUC_last_ values were 5.60 ± 1.30, 6.20 ± 2.01, and 7.43 ± 3.81 ng·h/mL for 25, 50, and 100 mg/kg YRA-1909, respectively. CA-3-G and CA-4-G were formed from CA, as shown in Figure 1 [12,13,14,15,16] and their dose-normalized (based on 25 mg/kg YRA-1909) AUC_last_ values were 111.15 ± 37.32, 104.39 ± 40.23, and 101.33 ± 47.14 ng·h/mL for CA-3-G and 6.04 ± 0.77, 7.47 ± 2.02, and 6.40 ± 2.71 ng·h/mL for CA-4-G, respectively. AUC_last_ and C_max_ values of CA-3-G were higher than those for CA and CA-4-G, which suggests that CA-3-G may be a major metabolite after oral administration of YRA-1909. FA, which was formed via methylation of CA [12,13,14,15], was detected 0.083 or 0.25 h after oral administration of YRA-1909. After oral administration of YRA-1909, the AUC_last_ values of the metabolites, CA, CA-3-G, and CA-4-G were higher than those of CGA, CCGA, and NCGA due to extensive metabolism, which were similar to the metabolism of CGA, CCGA, and NCGA in humans and rats [12,14,15]. 

Repeated administration of YRA-1909 at 50 mg/kg for seven days to male SD rats did not affect the plasma concentrations and pharmacokinetic parameters of any compounds when compared to those that received single administrations (Figure 4, Table 4).

## 5. Conclusions

A selective and sensitive LC-MS/MS method using liquid–liquid extraction as sample clean-up procedure was developed and validated for the simultaneous determination of CGA, CCGA, NCGA, and their metabolites, such as CA, CA-3-G, CA-4-G, and FA in rat plasma. The method was successfully applied to characterize the pharmacokinetics of CGA, CCGA, NCGA, CA, CA-3-G, CA-4-G, and FA after oral administration of YRA-1909 at 25, 50, and 100 mg/kg doses to male SD rats. These results constitute useful information for the development of YRA-1909 as a new herbal medicine for the treatment of rheumatoid arthritis, based on the evaluation of the pharmacokinetic properties of its main components (i.e., CGA, CCGA, and NCGA) and their active metabolites (CA, CA-3-G, and CA-4-G).

## Figures and Tables

**Table 1:** Linearity, LLOQ, intra-day and inter-day accuracy (RE, %) and precision (CV, %) of CGA, NCGA, CCGA, CA, CA-3-G, CA-4-G, and FA in rat plasma quality control (QC) samples.

Analytes	Concentration Range (ng/mL), Linear Equation ^a^, Linearity (*r*^2^) ^b^, LLOQ (ng/mL)	QC Concentration (ng/mL)	Intra-day (*n* = 5)		Inter-day (*n* = 15)	
			RE (%)	CV (%)	RE (%)	CV (%)
CGA	0.5–200	0.5	12.0	8.9	2.0	11.8
	*y* = 0.01110*x* − 0.00054	1.5	−6.7	6.4	−4.7	11.2
	0.9981	25	2.0	3.7	−1.9	10.5
	0.5	160	10.2	2.4	0.1	11.8
NCGA	0.5–200	0.5	−2.0	4.1	−2.0	8.2
	*y* = 0.00330*x* − 0.00039	1.5	−10.0	3.0	−1.3	10.1
	0.9983	25	0.7	3.1	2.8	8.0
	0.5	160	10.4	2.0	2.0	12.5
CCGA	2.5–1000	2.5	16.0	7.2	3.6	11.6
	*y* = 0.00149*x* − 0.00068	7.5	−6.3	4.8	−3.9	6.7
	0.9974	125	3.0	4.2	2.8	5.6
	2.5	800	−4.4	3.2	−1.9	8.3
CA	2.5–1000	2.5	−8.8	7.9	−2.8	18.1
	*y* = 0.03784*x* − 0.00235	7.5	−3.9	8.3	−2.8	8.6
	0.9959	125	6.5	5.0	6.5	6.2
	2.5	800	4.1	5.8	0.9	9.2
CA-3-G	2.5–1000	2.5	0.8	7.1	−0.4	8.0
	*y* = 0.00174*x* + 0.00022	7.5	−8.3	4.2	−5.9	9.6
	0.9981	125	4.3	2.4	2.4	6.2
	2.5	800	8.8	5.1	0.9	10.4
CA-4-G	2.5–1000	2.5	−6.8	10.7	−5.2	9.3
	*y* = 0.00140*x* + 0.00031	7.5	−7.2	7.0	0.3	11.6
	0.9977	125	10.0	3.5	6.7	6.4
	2.5	800	7.7	4.7	1.2	9.6
FA	12.5–5000	12.5	12.6	8.5	9.0	7.7
	*y* = 0.00628*x* − 0.02194	37.5	−3.2	7.2	−6.5	7.7
	0.9969	625	−3.0	3.8	−7.4	6.0
	12.5	4000	4.8	3.9	−0.1	9.6

^a^*y*: peak area ratio, *x*: concentration; ^b^*r*^2^: coefficients of determination.

**Table 2:** Matrix effects and recoveries of CGA, NCGA, CCGA, CA, CA-3-G, CA-4-G, FA, and FA-d_3_ (IS) using six different rat plasma (*n* = 6).

Compounds	Nominal Concentration (ng/mL)	Matrix Effect ^a^ (%)		Recovery ^b^ (Mean ± SD, %)
		Mean	CV (%)	
CGA	1.5	91.6	7.6	63.0 ± 6.3
	25	92.0	11.0	71.7 ± 6.9
	160	87.1	5.4	71.9 ± 9.4
NCGA	1.5	96.1	3.6	41.4 ± 4.0
	25	90.8	3.2	43.4 ± 3.7
	160	89.0	7.1	41.8 ± 1.4
CCGA	7.5	98.0	6.6	61.5 ± 5.2
	125	92.4	2.2	63.0 ± 7.8
	800	89.3	6.2	65.2 ± 5.7
CA	7.5	87.7	7.2	94.0 ± 9.9
	125	93.0	11.8	86.1 ± 10.4
	800	88.6	6.2	91.2 ± 6.3
CA-3-G	7.5	95.9	2.0	46.5 ± 4.3
	125	95.8	7.4	51.2 ± 3.4
	800	88.5	8.5	52.8 ± 4.2
CA-4-G	7.5	92.7	1.7	51.3 ± 6.0
	125	95.6	5.3	53.0 ± 3.4
	800	86.5	9.7	55.8 ± 4.2
FA	37.5	96.8	2.2	93.3 ± 8.7
	625	87.5	8.4	96.9 ± 8.7
	4000	97.5	8.6	97.4 ± 4.2
FA-d3	20	96.0	5.4	100.1 ± 2.0

^a^ Matrix effect expressed as the ratio of the mean peak area of an analyte spiked post-extraction to the mean peak area of same analyte standards multiplied by 100. ^b^ Recovery calculated as the ratio of the mean peak area of an analyte–spiked plasma prior to liquid–liquid extraction to the mean peak of an analyte spiked after liquid-liquid extraction of blank plasma multiplied by 100.

**Table 3:** Post-preparation, short-term, long-term and freeze-thaw stabilities of CGA, NCGA, CCGA, CA, CA-3-G, CA-4-G, and FA in rat plasma quality control samples (*n* = 3).

Analytes and Nominal Concentration (ng/mL)	Post-Preparative (24 h at 4 °C)		Short-Term (2 h on ice)		Long-Term (28 days at −80 °C)		Freeze-Thaw 3 Cycles (−80 °C to room temp.)	
	CV, %	RE, %	CV, %	RE, %	CV, %	RE, %	CV, %	RE, %
CGA								
1.5	8.8	−9.3	2.3	−12.0	7.0	−14.0	6.2	−14.0
160	2.1	9.4	2.5	−5.4	4.1	−12.3	3.2	−10.0
NCGA								
1.5	14.4	−2.7	2.3	−12.0	3.7	−9.3	10.1	−14.0
160	2.7	11.2	4.2	−5.9	5.6	−6.3	5.9	−5.3
CCGA								
7.5	5.1	−3.2	3.7	−14.3	5.9	−8.9	7.6	−11.9
800	1.3	5.5	2.9	−4.6	4.4	−14.4	3.0	−9.4
CA								
7.5	4.1	−0.1	9.0	−12.4	7.3	−6.3	8.3	−7.2
800	0.9	10.6	2.1	−7.7	7.6	−12.7	2.1	−11.0
CA-3-G								
7.5	9.9	−10.0	6.1	−14.6	7.3	−7.1	9.1	−10.5
800	1.1	9.1	2.8	−1.5	2.2	−14.8	4.4	−11.9
CA-4-G								
7.5	6.2	−9.6	8.1	−9.3	7.9	1.6	4.7	−3.6
800	1.0	10.9	3.2	−0.4	2.1	−13.9	4.7	−9.4
FA								
37.5	3.2	−7.0	5.4	−11.2	5.9	−11.0	5.6	−7.7
4000	4.6	−8.6	2.7	−4.8	6.1	−8.8	3.9	−8.4

**Table 4:** Pharmacokinetic parameters of CGA, CCGA, NCGA, CA, CA-3-G, and CA-4-G after a single oral administration of YRA-1909 at doses of 25, 50, and 100 mg/kg (equivalent to 1.2, 2.4, and 4.8 mg/kg CGA; 0.275, 0.55, and 1.10 mg/kg NCGA; 0.87, 1.74 and 3.48 mg/kg CCGA) and repeated administration of 50 mg/kg YRA-1909 for seven days to male rats (mean ± SD).

Compounds	PK Parameters	Dose of YRA-1909 (mg/kg)			
		Single Oral Dosing			7-Day Repeated Oral Dosing
		25 (*n* = 6)	50 (*n* =12)	100 (*n* = 6)	50 (*n* = 6)
CGA	C_max_ (ng/mL)	3.11 ± 0.96	5.59 ± 1.61	8.73 ± 2.83	4.85 ± 1.25
	T_max_^1^ (h)	0.25 (0.083–0.25)	0.25 (0.083–0.5)	0.25	0.25 (0.083–0.5)
	AUC_last_ (ng∙h/mL)	4.29 ± 1.92	8.16 ± 3.65	14.38 ± 5.06	7.54 ± 1.86
	t_1/2_ (h)	NC	NC	1.52 ± 0.79	NC
	MRT (h)	1.19 ± 0.22	1.48 ± 0.50	1.67 ± 0.63	1.48 ± 0.18
CCGA	C_max_ (ng/mL)	NC	4.01 ± 0.82	5.64 ± 1.45	3.81 ± 0.72
	T_max_^1^ (h)	NC	0.25 (0.083–0.5)	0.25 (0.083–0.25)	0.25 (0.083–0.5)
	AUC_last_ (ng∙h/mL)	NC	4.15 ± 4.10	6.26 ± 4.70	3.35 ± 3.42
	MRT (h)	NC	0.61 ± 0.55	0.84 ± 0.65	0.56 ± 0.57
NCGA	C_max_ (ng/mL)	0.70 ± 0.12	1.18 ± 0.26	1.69 ± 0.43	1.08 ± 0.28
	T_max_^1^ (h)	0.17 (0.083–0.5)	0.25 (0.083–0.5)	0.25 (0.083–0.25)	0.25 (0.083–0.25)
	AUC_last_ (ng∙h/mL)	0.81 ± 0.22	1.61 ± 0.90	3.40 ± 1.97	1.60 ± 0.79
	MRT (h)	0.68 ± 0.30	1.00 ± 0.58	1.66 ± 0.91	1.09 ± 0.55
CA	C_max_ (ng/mL)	3.92 ± 1.47	7.31 ± 2.12	16.72 ± 5.45	6.07 ± 0.94
	T_max_^1^ (h)	1.25 (1.0–1.5)	0.25 (0.083–1.0)	0.25 (0.25–2.0)	0.17 (0.083–2.0)
	AUC_last_ (ng∙h/mL)	5.60 ± 1.30	12.40 ± 4.03	29.73 ± 15.24	11.75 ± 3.31
	MRT (h)	0.90 ± 0.11	1.29 ± 0.31	1.74 ± 0.87	1.26 ± 0.33
CA-3-G	C_max_ (ng/mL)	69.09 ± 25.06	129.05 ± 30.41	238.55 ± 97.46	132.18 ± 26.70
	T_max_^1^ (h)	0.38 (0.25–1.0)	0.38 (0.25–1.0)	0.25 (0.25–1.0)	0.38 (0.25–1.0)
	AUC_last_ (ng∙h/mL)	111.15 ± 37.32	208.77 ± 80.46	405.33 ± 188.56	216.38 ± 25.06
	t_1/2_ (h)	1.05 ± 0.38	0.93 ± 0.23	0.82 ± 0.23	0.88 ± 0.19
	MRT (h)	1.49 ± 0.52	1.33 ± 0.24	1.68 ± 0.69	1.33 ± 0.12
CA-4-G	C_max_ (ng/mL)	3.86 ± 0.94	6.97 ± 1.91	9.38 ± 2.56	7.77 ± 1.85
	T_max_^1^ (h)	1.0 (1.0–1.5)	0.75 (0.25–2.0)	1.0 (0.5–2.0)	1.5 (1.0–2.0)
	AUC_last_ (ng∙h/mL)	6.04 ± 0.77	14.95 ± 4.03	25.59 ± 10.84	18.95 ± 3.30
	MRT (h)	1.01 ± 0.18	1.48 ± 0.27	1.77 ± 0.46	± 0.23

^1^ T_max_ presented median value with the range in parentheses. NC: Not calculable.
